# P-glycoprotein expression skews mitochondrial dye measurements in T cells

**DOI:** 10.3389/fimmu.2025.1560104

**Published:** 2025-06-18

**Authors:** Sophia P. M. Sok, Jianan Cheng, Klaudia Strucinska, Narcis I. Popescu, Lihua Wu, Qiuqing Ke, William B. Kiosses, David Stanford, Willard M. Freeman, Satoshi Matsuzaki, Tommy L. Lewis, Meng Zhao

**Affiliations:** ^1^ Arthritis and Clinical Immunology Research Program, Oklahoma Medical Research Foundation, Oklahoma, OK, United States; ^2^ Aging and Metabolism Research Program, Oklahoma Medical Research Foundation, Oklahoma, OK, United States; ^3^ Division of Inflammation Biology, La Jolla Institute for Immunology, La Jolla, CA, United States; ^4^ Center for Biomedical Data Science, Oklahoma Medical Research Foundation, Oklahoma, OK, United States; ^5^ Genes and Human Disease Research Program, Oklahoma Medical Research Foundation, Oklahoma, OK, United States; ^6^ Departments of Biochemistry & Molecular Biology, Neuroscience and Physiology, University of Oklahoma Health Sciences Center, Oklahoma, OK, United States; ^7^ Department of Microbiology and Immunology, University of Oklahoma Health Sciences Center, Oklahoma, OK, United States; ^8^ Stephenson Cancer Center, University of Oklahoma Health Sciences Center, Oklahoma, OK, United States

**Keywords:** T cells, mitochondria, oxidative phosphorilation, mitotracker, TMRE, P-glycolprotein

## Abstract

Assays to monitor metabolic parameters of immune cells at a single cell level provide efficient means to study immunometabolism. We show here that staining intensity of mitochondria targeting probes in T cells is dramatically influenced by P-glycoprotein/P-gp expression, a xenobiotic efflux pump that extrudes these fluorescent dyes. Discrepancies between MitoTracker Green FM/MTG signals and multiple dye-independent measurements are seen in CD4 T and CD8 T cell subsets and are corrected by P-gp inhibition (PSC833) during MTG staining. We further investigate invariant Natural Killer T (iNKT) cells, which express the highest level of P-glycoprotein among T cells. Using mtDNA abundance, mitochondrial volume, respiration and proteomics, we establish that iNKT cells have higher mitochondrial content and activity than CD4 T cells, opposite to what MTG signals reveal. A similar phenomenon is also seen in human PBMCs, and with TMRE, a dye indicator of mitochondrial membrane potential. Collectively, these data argue that P-glycoprotein expression is a significant confounding factor when analyzing T cells using mitochondrial specific dyes. Complementary methods are necessary to reliably assess mitochondrial features in T cells.

## Introduction

Metabolic reprogramming regulates the dynamic changes in T cell activation states, effector functions and tissue distribution according to environmental cues. In particular, mitochondria actively adapt to the cellular metabolic demands, and change their quantity, quality and activity to modulate T cells (1). Multiple mitochondria specific dyes have traditionally been used to profile mitochondrial properties in immune cells using flow cytometry. For instance, MitoTrackerGreen FM (MTG) is widely used to reflect mitochondrial mass as it reacts with free thiol groups of cysteine residues of mitochondrial proteins regardless of mitochondrial polarization status (2). Therefore, MTG is generally considered a mitochondrial membrane potential (ΔΨm) insensitive indicator. However, several studies have shown that ΔΨm and redox changes may influence MTG staining (3–5). Tetramethylrhodamine ethyl ester/Tetramethylrhodamine methyl ester (TMRE/TMRM) are lipophilic cationic dyes that are attracted to the negative potential across the plasma membrane, and subsequently across the mitochondrial inner membrane, to preferentially accumulate into mitochondria. Thus, their staining intensities are routinely used to report mitochondrial membrane potential (6, 7). Although these dyes may gain mitochondrial specificity through different means, they are cell permeant. Divergence in dye staining intensity among different T cell populations, or under varying conditions, is typically viewed as evidence of changes in mitochondrial network.

P-glycoprotein 1 (P-gp, also known as Multidrug resistance-1, Mdr1; encoded by Abcb1a and Abcb1b in mice and ABCB1 in humans) is a plasma membrane transporter that pumps xenobiotics out of cells in an ATP dependent manner (8). It is highly expressed in many cancers and mediates drug resistance (9). MTG and tetramethylrhodamine (TRM) are substrates for P-gp (10, 11). As TMRE and TMRM are both ester derivatives of TRM, they may be extruded by P-gp as well. P-gp is also expressed in healthy tissues (9). Interestingly, it was shown in a P-gp reporter mouse that T cell populations express different levels of P-gp (12). Therefore, we tested the hypothesis that comparison of mitochondrial properties between different T cell populations based on fluorescent dye signals may lead to erroneous conclusions due to P-gp expression levels.

## Method

### Mice

All mouse experiments were approved by Institutional Animal Care and Use Committee at the Oklahoma Medical Research Foundation. The C57BL/6J (B6) (000664) and B6;129S-Gt(ROSA)26Sortm1(CAG-COX8A/Dendra2)Dcc/J (018385) mice were purchased from The Jackson Laboratory.

### Human PBMCs

Studies on peripheral blood mononuclear cells (PBMCs) were conducted in accordance with the Declaration of Helsinki and approved by the Institutional Review Board at the Oklahoma Medical Research Foundation. Adult volunteers, both males and females, were enrolled and provided written informed consent before each blood draw. PBMCs were isolated by density gradient centrifugation using Histopaque-1077 (Millipore-Sigma), washed with RPMI-1640 basal media, and processed for flow cytometry as described below.

### Antibodies and reagents

The antibodies conjugated with various fluorophores were from BioLegend: anti-mouse CD19 (1D3), B220 (RA3-6B2), CD8a (53-6.7), CD4 (GK1.5/RM4-5), TCRβ (H57-597), ICOS (C398.4A), CD62L (MEL-14), CD44 (IM7), CD25 (PC61), and anti-human-CD19 (HIB19), CD14 (HCD14), CD3ϵ (OKT3), CD4 (SK3), as well as CD8a (SK1). The biotinylated antibodies for iNKT cell enrichment includingTER-119 (TER-119), B220 (RA3-6B2), CD19 (6D5), CD8a (53-6.7), CD11b (M1/70), CD11c (N418), F4/80 (BM8), Ly-6G/Ly-6C (RB6-8C5), and γδTCR (GL3), CD62L (MEL-14), and CD24 (M1/69) Abs, as well as the anti-ARTC2.2 (s+16a) nanobody were from Biolegend. Human and mouse CD1d/PBS-57 tetramers were obtained from the NIH Tetramer Core Facility.

The T cell culture medium, DMEM, and its supplements penicillin/streptomycin, sodium pyruvate, nonessential amino acids, HEPES, 2-mercaptoethanol, as well as live death blue cell stain kit was from ThermoFisher. FBS was from R&D. The 7-aminoactinomycin D (7-AAD) was obtained from BD Biosciences.

PSC833 was from Santa Cruz Biotechnology. Poly-L-ornithine solution (0.1mg/ml) was from ThermoFisher. Fluoromount-G was obtained from Southern Biotech.

### Flow cytometry and dye staining

Single cell suspensions were stained with live death blue stain prior to surface antibodies. For dye staining, the cells were then pretreated with or without 1μM PSC 833 in cell culture medium for 10 min prior to addition of mitochondrial dyes, 10nM (final concentration) MitoTracker green FM or 5 nM TMRE (Invitrogen) and incubation for 15 min.

### Enrichment and isolation of T cells

For all cell sorting experiments, mice were i.v. injected with 50ug anti-ARTC2.2 (s+16a) nanobody before sacrificing (13). iNKT cells were negatively enriched using biotinylated anti-mouse Ter119, B220, CD19, CD8a, CD11b, CD11c, F4/80, Ly-6G/Ly-6C, γδTCR (GL3), CD62L and MojoSort Streptavidin Nanobeads (BioLegend). iNKT were sorted as 7-AAD^-^TCRβ^int^CD1dtet^+^ cells; naïve CD4 T cells were sorted as 7-AAD^-^CD1dtet^-^TCRβ^+^ CD4^+^CD25^-^CD62L^+^.

### Proteomics

Freshly sorted naïve CD4 T and iNKT were processed as described previously (14). Briefly, the cells were washed in PBS once and lysed with protein lysis buffer (150 mM sodium chloride, 1% NP-40, 0.5% sodium deoxycholate, 0.1% sodium dodecyl sulfate, 50 mM Tris, pH7.4) with 1 × Halt Protease and Phosphatase Inhibitor cocktail (Thermo Scientific). Proteomic profiling of the protein lysates was performed by the IDeA National Resource for Quantitative Proteomics. The heatmaps of the differentially expressed proteins in CD4 T and iNKT cells were generated using the free web server tool, Heatmapper (http://www.heatmapper.ca/expression). The row Z-score, a scaling method for visualization in heatmap, was shown. The proteomics data is in the process of being deposited at Zenodo with pending accession number.

### Mitochondrial DNA quantification by real-time PCR

The thymic and splenic CD4 T and iNKT cells were sorted from C57BL/6J mice. The genomic DNA and mitochondrial DNA were extracted with DNeasy Blood & Tissue (QIAGEN) according to the manufacturer’s protocol. The real-time PCR was performed using Universal SYBR Green fast qPCR master mix (ABclonal) with respective primers in a LightCycler 480 Instrument II (Roche). The relative mitochondrial DNA/nuclear DNA (mtDNA:nDNA) ratio was calculated using the ΔΔCt method.

mt-CO1_Fwd: GCCCCAGATATAGCATTCCC;

mt-CO1_Rev: GTTCATCCTGTTCCTGCTCC;

mtND1_Fwd: CTAGCAGAAACAAACCGGGC;

mtND1_Rev: GTATGGTGGTACTCCCGCTG;

Cytb_Fwd: GCCACCTTGACCCGATTCT;

Cytb_Rev: TTCCTAGGGCCGCGATAAT;

Tert_Fwd: CTAGCTCATGTGTCAAGACCCTCTT;

Tert_Rev: GCCAGCACGTTTCTCTCGTT.

### mtDNA sequencing and analysis

mtDNA was amplified using 1 ng total gDNA and long-range PCR (TaKaRa LR DNA Polymerase) with primers specific for the mouse mitochondrial genome

(FWD: AAACGAAAGTTTGACTAAGTTATACCTCTTAGGGTTGGT and

REV: TGGGAACTACTAGAATTGATCAGGACATAGGGTTTGATAG) with the following hot-start PCR reaction conditions; 94°C for 5 minutes, 35 cycles of 94°C for 15 seconds, 67°C for 11 minutes, and a final extension step of 72°C for 10 minutes. DNA amplicons were determined to be of correct size by Agilent TapeStation. 500 pg of mtDNA amplicon per sample were used for sequencing library preparation using Nextera XT reagents according to manufacturer’s instructions (Illumina) as previously described (15). The average size of libraries was quantified using the High Sensitivity dsDNA assay reagents and the Bioanalyzer 2100 (Agilent). Molar concentrations of libraries were determined by standard-curve qPCR according to manufacturer’s instructions (KAPA Biosystems). Libraries were diluted to 2 nM and pooled for benchtop sequencing (MiSeq, Illumina) using paired-end (2x75bp) reagents (Illumina) at a final library concentration of 12 pM. Paired-end sequencing reads were de-multiplexed and imported for analysis in CLC Genomics Workbench 22.0.2 (Qiagen). Trimmed reads were then aligned to the annotated mouse mitochondrial genome (NCBI accession NC_005089.1, 16299bp) using the Large Gap Read Mapping plug-in tool. Variants were determined from reads maps using the Low Frequency Variant Detection tool. The resulting identified variants were used for analysis of variant rates (frequency/variant).

### Confocal microscopy analysis

The sorted CD4 T, as well as iNKT cells from Dendra2 transgenic mice were seeded in 96-well glass bottom plates (Cellvis; 0.170 ± 0.005mm) coated with poly-L-ornithine (0.1mg/ml) and the cells were fixed with fixative buffer (2% paraformaldehyde with 0.075% glutaraldehyde in PBS) for 10 min prior to mounting with Fluoromount-G. The Z-stacked images of the mitochondrial were acquired using a Zeiss LSM 880 Confocal Laser Scanning microscope with airyscan using a 100X oil immersion objective with 5X zoom. The images were processed using the Airyscan Processing features in the Zen Black software, then transferred to Imaris software (version 9.3.1) for 3D rendering and analysis. An iso-surface was created around Dendra2 green fluorescence signal with the seed points diameter of 0.3 μm (size of an individual mitochondria based on EM data) was used.

### Seahorse cell mito stress test

Cellular mitochondrial stress test was performed on Seahorse XF96 extracellular flux analyzer (Agilent, Santa Clara, CA) according to the manufacturer’s instructions. Mouse iNKT cells and CD4 T cells were plated on a sterile XF96 plates at 3 × 10 (5) cells per well. The XF96 plates were pre-treated with Poly-L-ornithine (0.1mg/ml) for one hour at room temperature, washed once with water, and air-dried. The metabolic profiles of the plated cells were sequentially measured as baseline OCR, ATP-coupled respiration after addition of oligomycin (Oligo, 2.5 μM), maximal uncoupled respiration after stimulation with DNP (150 μM), and non-mitochondrial respiration after addition of rotenone (R) (2 μM) and antimycin A (A) (2 μM).

## Results

### P-glycoprotein expression affects mitochondrial dye measurements in T cells

**Figure 1:**
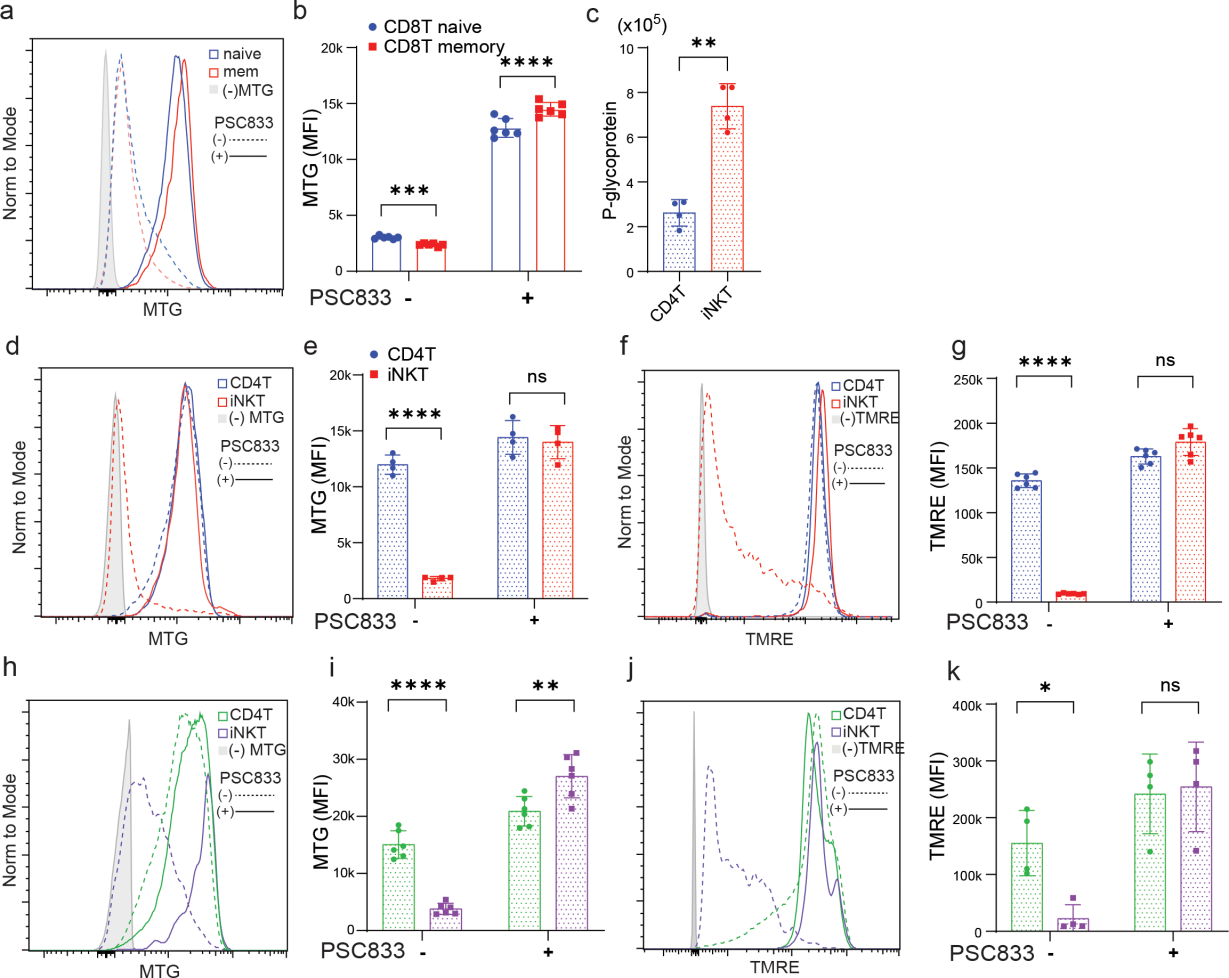
P-glycoprotein (P-gp) expression affects mitochondrial dye measurements in T cells. **(a, b)** MTG signals from splenic naïve (CD44^-^CD62L^+^) and memory (CD44^+^CD62L^+^) CD8 T cells stained with or without PSC833 (1uM). **(c)** Protein expression level of P-glycoprotein based on Variance Stabilization Normalization (VSN) Normalized Intensities from proteomic analysis. **(d, e)** MTG and **(f, g)** TMRE signals from thymic CD4 T (CD8a^-^CD1d-aGC-tetramer^-^ CD4^+^TCRβ^hi^) and iNKT (CD8a^-^ CD1d-aGC-tetramer^+^ TCRb^int^) cells stained with or without PSC833 as in **(a)**. **(h, i)** MTG and **(j, k)** TMRE signals from PBMC CD4 T (CD8a^-^CD1d-aGC-tetramer^-^ CD4^+^TCRb^hi^) and iNKT (CD8a^-^ CD1d-aGC-tetramer^+^ TCRb^int^) cells stained with or without PSC833 as in **(a)**. The data shown in **(a, b, d-k)** are one representative experiment out of three independent experiments. The data shown in c are from samples collected from two sorting experiments and proteomes analyzed together. ns, non-significant, *p < 0.05, **p < 0.01, ***p < 0.001, ****p < 0.0001, paired Student’s t test.

An Abcb1a-knockin reporter allele (*Abcb1a^AME^*) (12) showed that T cell populations express different levels of P-gp, and memory T cells have higher levels of reporter expression than naïve T cells (12). Consistently, mouse memory CD4 T and CD8 T cells showed a lower MTG signal than their naïve counterparts (**Figures 1a, b**; **Supplementary Figures S1a, b**). However, it has been reported that maximal and spare respiratory capacity, and mitochondrial DNA relative to nuclear DNA are higher in memory T cells (16). Similar discrepancies have been observed in hematopoietic stem cells and was found to be due to dye efflux through P-gp (8). Next, we stained the cells with MTG in the presence of P-gp inhibitor PSC833. MTG signal was drastically increased, and memory T cells now exhibited higher MTG fluorescence than naïve T cells (**Figures 1a, b**; **Supplementary Figures S1a, b**), indicating that higher dye efflux mediated by P-gp in memory T cells leads to artificially lower MTG signals.

A group of T cells co-expressing NK1.1 was found to express the highest level of Abcb1a reporter compared to other T cells at steady state (12), suggesting that MTG staining may not reflect mitochondrial content in this T cell population. The majority of NK1.1^+^ T cells are invariant Natural Killer T cells (iNKT cells), expressing invariant TCRα chain encoded by Vα14-Jα18 gene segments paired with a limited repertoire of TCRβ chains in mice and humans (17). Proteomic analysis of freshly isolated mouse T cells confirmed that iNKT cells expressed significantly higher P-gp than naïve CD4 T cells (**Figure 1c**). As with memory T cells, MTG signals were much lower in iNKT cells compared to that in CD4 T cells (**Figures 1d, e**) in line with previous publications (18, 19). Interestingly, in the presence of PSC833, MTG signals were comparable in iNKT and CD4 T cells (**Figures 1d, e**). Likewise, TMRE signals were much lower in iNKT cells in the absence of PSC833, and P-gp inhibition increases TMRE fluorescence in iNKT cells to the level observed in CD4 T cells (**Figures 1f, g**), suggesting that TMRE is also a substrate of P-gp mediated efflux. Further, we compared iNKT cells and CD4 T cells from fresh human peripheral blood mononuclear cells (PBMCs) and observed parallel changes in MTG and TMRE staining signals before and after P-gp blockade as in mice (**Figures 1h-k**), suggesting high P-gp expression is a conserved feature in iNKT cells. Overall, these data indicate that P-gp in iNKT cells extrudes MTG and TMRE making them poor indicators of mitochondrial traits in these cells.

### Mitochondrial proteome supports high oxidative phosphorylation and mitochondrial metabolism in iNKT cells

**Figure 2:**
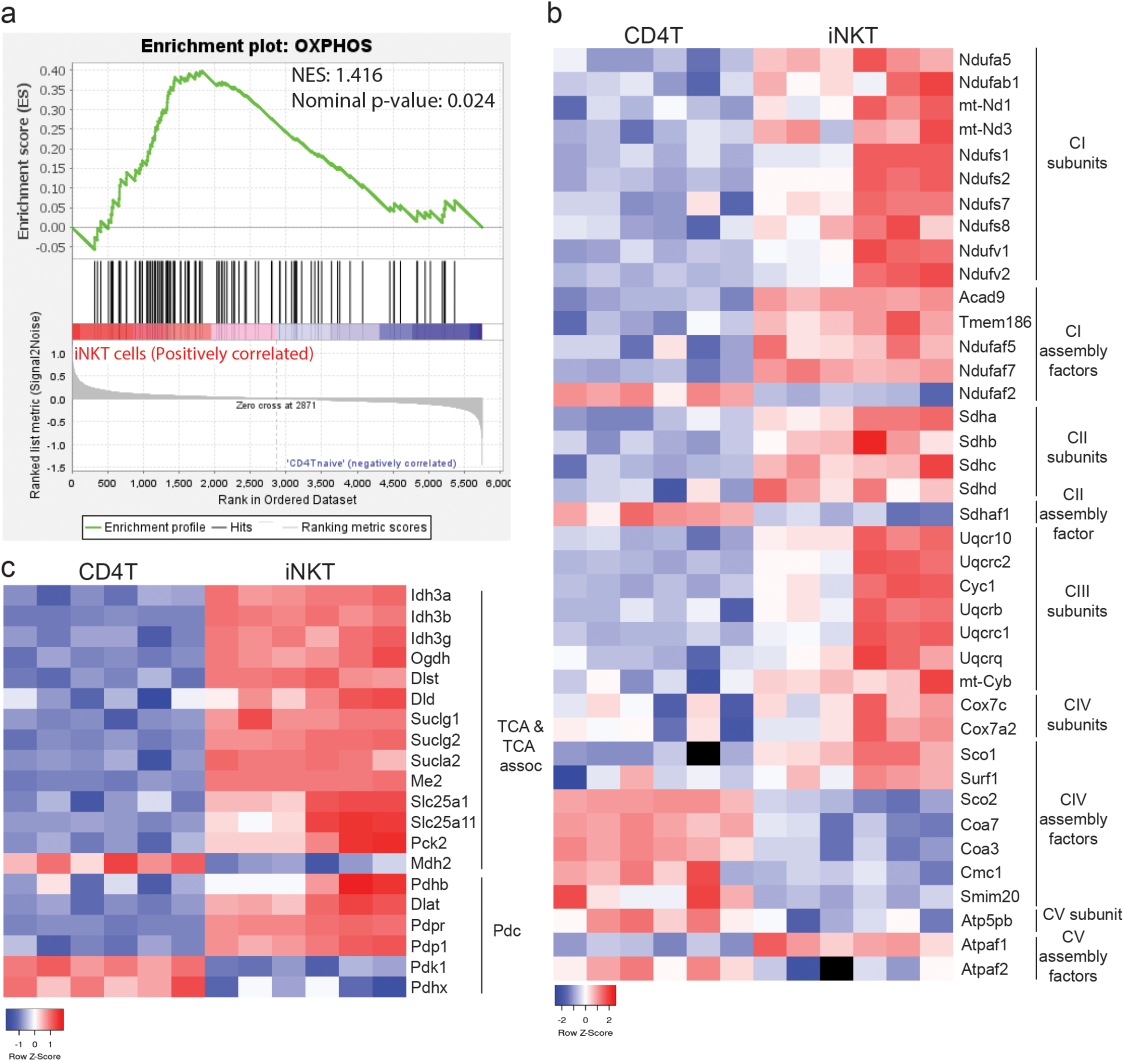
Mitochondrial proteome supports high oxidative phosphorylation and mitochondrial metabolism in iNKT cells. **(a)** Gene set enrichment analysis of 107 oxidative phosphorylation related proteins comparing the proteomes of thymic iNKT cells and CD4 T cells. **(b, c)** Differentially expressed Electron Transport Chain (ETC) complex proteins **(b)**, TCA cycle related and pyruvate dehydrogenase complex proteins **(c)**.

To determine if corrected MTG florescence upon P-gp inhibition faithfully reports mitochondrial mass, we next compared expression levels of mitochondrial proteins. Total proteins extracted from freshly isolated thymic iNKT cells and CD4 T cells were subjected to proteomic analysis. Gene set enrichment analysis (GSEA) against 149 mitochondrial pathways curated by MitoCarta3.0 (20) showed that pathways related to electron transport chain subunits or oxidative phosphorylation were significantly enriched at nominal p value < 0.05 and positive normalized enrichment scores (NES) correlating with iNKT cells (**Figure 2a**). Electron transport chain (ETC) located in the inner mitochondrial membrane passes electrons from NADH and FADH2 to oxygen, generating a proton gradient across the mitochondrial membrane to fuel the phosphorylation of ADP to ATP (21). Among ETC complex I (NADH ubiquinone oxidoreductase; CI) proteins detected (49 out of 66), 15 proteins showed significantly differential expression comparing iNKT cells and CD4 T cells, which included 10 CI subunits and 5 CI assembly factors (**Figure 2b**). All but one protein (NADH:ubiquinone oxidoreductase complex assembly factor 2; Ndufaf2) were more abundant in iNKT cells. Among ETC complex II (succinate dehydrogenase; CII) detected proteins (5 out of 8), all four subunits, succinate dehydrogenase (SDHA), succinate dehydrogenase [ubiquinone] iron–sulfur subunit mitochondrial (SDHB), succinate dehydrogenase complex subunit C (SDHC), and succinate dehydrogenase complex subunit D (SDHD), were more abundant in iNKT cells (**Figure 2b**). Similarly, all 7 proteins of ETC complex III (cytochrome bc1 complex; CIII) that were differentially expressed were more abundant in iNKT cells. For ETC complex IV (cytochrome c oxidase; CIV) and V (ATP synthase, CV) proteins, the expression patterns in iNKT cells and CD4 T cells were also distinct. Overall, among 167 ETC complex proteins (20), 95 proteins were detected, and 39 proteins showed differential expression (DE). Among the DE proteins, 77% (30 out of 39) were expressed at a higher level in iNKT cells (**Figure 2b**), suggesting that oxidative phosphorylation at a steady state is more active in iNKT cells.

The tricarboxylic acid cycle (TCA cycle) occurring in the mitochondrial matrix consumes acetyl-CoA generated from catabolism of carbohydrates, lipids and proteins to produce NADH and FADH2, which in turn is used by oxidative phosphorylation as above to generate ATP (22). The cycle reactions are carried out by eight enzymes, out of which, aconitase (Aco2), fumarate hydratase (Fh) and citrate synthase (Cs) were not differentially expressed in iNKT and CD4 T cells (data not shown). Except for malate dehydrogenase (Mdh2), all other enzyme complexes, isocitrate dehydrogenase subunits (Idh3a, Idh3b and Idh3g), α-ketoglutarate dehydrogenase subunits (Ogdh, Dlst and Dld), succinyl-CoA synthetase subunits (Sucg1, Sucg2 and Suca2), and succinate dehydrogenase subunits (**Figure 2b**) were all expressed at higher level in iNKT cells (**Figure 2c**). Transporters that exchange TCA cycle intermediates with cytosolic metabolites (Slc25a1, Slc25a11), or enzymes that convert TCA cycle intermediates to alternative metabolites (Me2, Pck2), were also more abundant in iNKT cells (**Figure 2c**). These data indicate TCA cycle could be more active in iNKT cells than in CD4 T cells under homeostasis.

Pyruvate dehydrogenase complex (Pdc) is an important enzyme that converts pyruvate from glycolysis into acetyl-CoA (23). While the non-catalytic subunit, pyruvate dehydrogenase complex component X (Pdhx) was expressed higher in CD4 T cells, the catalytic subunits, pyruvate dehydrogenase E1 subunit beta (Pdhb), dihydrolipoamide S-acetyltransferase (Dlat; E2), and dihydrolipoamide dehydrogenase (Dld; E3; also a α-ketoglutarate dehydrogenase subunit above) were expressed higher in iNKT cells (**Figure 2c**). Furthermore, Pdc is inhibited by pyruvate dehydrogenase kinase (Pdk1) through serine phosphorylation on E1, while pyruvate dehydrogenase phosphatase (Pdp) dephosphorylates E1 thereby reinstating the complex activity. Interestingly, Pdk1 was expressed at a lower level, whereas Pdp1 and pyruvate dehydrogenase phosphatase regulatory subunit (Pdpr) were higher in iNKT cells (**Figure 2c**). These data suggest that pyruvate dehydrogenase activity is elevated in iNKT cells to potentially supply pyruvate-derived acetyl-CoA as a main fuel for mitochondrial metabolism.

### iNKT cells exhibit high mitochondrial content and activity opposite to low MTG signal

**Figure 3:**
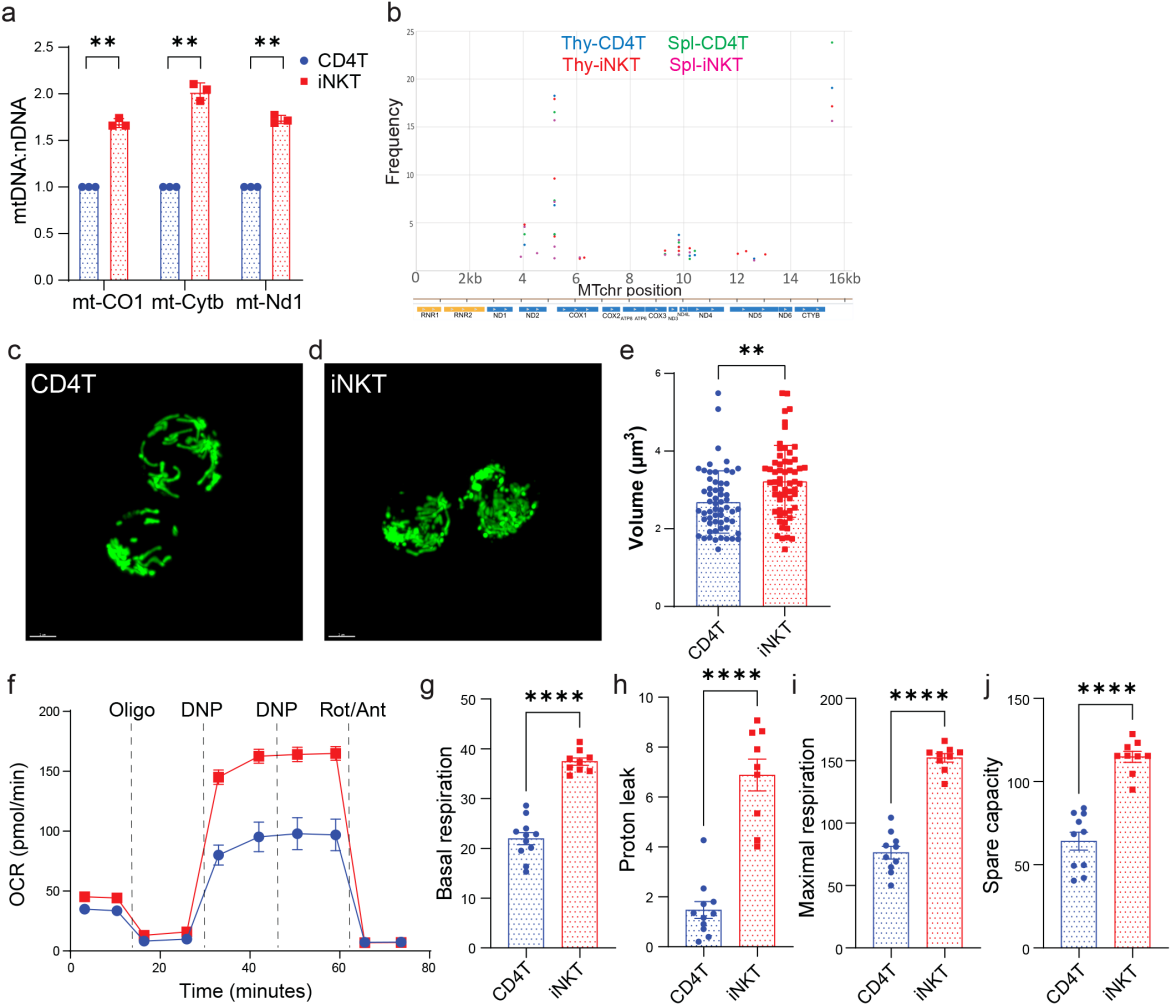
iNKT cells exhibit high mitochondrial content and activity. **(a)** Quantification of mitochondrial genome encoded genes relative to nuclear DNA (nDNA) in freshly isolated thymic T cells. **(b)** Ideogram of the 16,299 basepair (bp) mtDNA genes and annotations, and the variant frequencies plotted at each variant locus identified from thymic and splenic CD4 T cells and iNKT cells. **(c, d)** Confocal imaging of freshly isolated thymic T cells from Dendra2 transgenic mice. Scale bars indicate 2μm. **(e)** Quantification of mitochondrial volume based on Dendra2 signals as in c and d. **(f)** Average oxygen consumption rate (OCR) in sorted thymic T cells measured in a Seahorse Mitostress test. **(g)** Basal OCR, **(h)** proton leak, **(i)** maximal OCR, and **(j)** spare respiratory capacity were calculated based on **(f)**. The data shown in a, c, d and f are one representative experiment out of three independent experiments. The data shown in e and g-j are pooled from three independent experiments. **p < 0.01, ****p < 0.0001, unpaired Student’s t test.

We next quantified mitochondrial DNA (mtDNA) relative to nuclear DNA in freshly isolated T cells. We tested three mitochondrial genome-encoded genes, cytochrome c oxidase I (mt-Co1), cytochrome b (mt-Cytb) and NADH dehydrogenase 1 (mt-Nd1). The levels of all three genes relative to nuclear DNA were higher in iNKT cells than in CD4 T cells from thymus and spleen (**Figure 3a**; **Supplementary Figure S1c**). Protein expressions of mt-Cytb and mt-Nd1 were also higher in iNKT cells (**Figure 2b**). High depth mitochondrial DNA sequencing revealed low frequencies of single nucleotide variants and the absence of large deletions in both iNKT cells and CD4T cells (**Figure 3b**), indicating that iNKT cells harbor wildtype mitochondrial genome. PhAM^floxed^ mice express a green fluorescent protein Dendra2 specifically localized to the mitochondrial matrix (24). We isolated T cells from these mice and examined mitochondrial volume based on Dendra2 signals using high resolution confocal microscopy. iNKT cells had higher total mitochondrial volume in thymus (**Figures 3c-e**) or spleen (**Supplementary Figures S1d-f**). Importantly, basal oxygen consumption rates (OCR), proton leak and maximal OCR in iNKT cells were higher than those in CD4 T cells from C57BL/6 thymus and spleen (**Figures 3f-j**; **Supplementary Figures S1g-k**), consistent with previous findings using thymic T cells from Balb/c mice (25), and elevated protein expression of ETC and TCA cycle components (**Figure 2**). Therefore, our data show that iNKT cells have higher mitochondrial content and activity than conventional CD4 T cells. Further, even in the presence of P-gp inhibitor, MTG fluorescence intensity alone may not be a reliable reporter comparing mitochondrial mass in immune cell populations with different P-gp expressions (**Figure 1d**, **Figure 3**). Dye independent methods are essential for evaluating mitochondria in immune cells.

## Discussion

Fluorescent probes to stain cells based on metabolic processes such as mitochondria are valuable tools and allow for convenient characterization of immunometabolism at a single cell resolution using flow cytometry. However, their intended uses may need to be thoroughly evaluated in immune cells. For example, 2NBDG, a fluorescent analogue of 2-deoxyglucose (2DG), has been recently reported to be a poor indicator of glucose uptake in T cells due to the specificity of glucose transporters (26). Regarding mitochondrial specific dyes, multiple factors play a role including cell size, cell shape, mitochondrial shape, inner membrane topology, metabolic state, and fluorescence quenching (27). The influence of P-glycoproteins on mitochondrial fluorescence readouts in T cell populations has not been explored to our knowledge.

P-glycoprotein/P-gp is an ATP dependent efflux pump on plasma membrane. It has also been shown to play physiological functions in dendritic cells, T cells and NK cells (12, 28–32), although it is best known for extruding chemotherapeutic drugs out of cancer cells (33). P-gp expression lowers the intracellular concentration of compounds such as nonyl acridine orange (NAO), dimethyloxadicarbocyanine iodide (DiOC2), rhodamine123, MTG and TMR, all of which preferentially stain mitochondria (27). Using a knockin genetic reporter, a recent study elegantly showed the expression profile of P-gp in hematopoietic cells (12). Among T cells, higher expressions were observed in the periphery (vs. thymus), CD8 T cells (vs. CD4 T), and memory T cells (vs. naïve T). iNKT cells (TCRβ^+^NK1.1^+^) express the highest level of P-gp among T cell subsets in thymus, spleen and small intestine lamina propria (12). As proof of principle, we observed lower MTG and TMRE signals in iNKT cells as compared to CD4 T and CD8 T cells (**Figure 1**, and data not shown). P-gp inhibitor (PSC833) treatment drastically elevated the fluorescence intensity in iNKT cells to that in CD4 T cells (**Figure 1**).

After TCR activation, iNKT cells increase the mRNA levels of TCA cycle enzymes to a greater extent than CD4 T cells, and oligomycin treatment inhibits iNKT cell proliferation, suggesting mitochondrial metabolism may be important for iNKT cell function (18). Deletion of Rieske iron-sulfur protein (RISP; *Uqcrfs*), an essential component of mitochondrial complex III, specifically abolishes iNKT cells, but not CD4 T and CD8 T cells, demonstrating the importance of mitochondrial metabolism during iNKT cell development (19). Contrary to what we have observed (25) (**Figure 3**; **Supplementary Figure S1**), iNKT cells were found to have reduced respiratory capacity and mitochondrial DNA (19). The main contributor to this discrepancy could be the source of iNKT cells: Vα14TgTCRα^−/−^ mice (19), wildtype BALB/c mice (25), and wildtype C57BL/6 mice (this study). We further show that many ETC complex components and TCA cycle enzymes are more abundant in iNKT cells, and that mitochondrial volume based on a mitochondrial targeted fluorescent protein Dendra2 is higher in iNKT cells (**Figures 2**, **3**). Altogether, our data unequivocally demonstrates that iNKT cells have higher mitochondrial content and activity at a steady state, consistent with the important role of this organelle during iNKT cell development and function. P-gp inhibition rescued partly the lower MTG staining intensity (**Figure 1**). Other transporters including Abcc1, Abcc3 and Abcg2 could also mediate dye efflux (34, 35), and are not inhibited by PSC833. Future studies should investigate the contribution of these additional efflux transporters, as well as new inhibitors of P-gp like Tariquidar (36).

Many factors have been reported to regulate P-gp expression in different cellular contexts (37). These include NF-kB, AP-1 (38), and PI3K/Akt/mTOR (39) pathway which are key for iNKT cell development and function (40–43). In cytotoxic T cells, Runx proteins, particularly Runx1 and Runx3, are required to maintain P-gp expression *in vivo (* 12). Runx3 occupies multiple cis-regulatory regions across the Abcb1a/Abcb1b locus in CTLs suggestive of a direct regulation (12). Both Runx1 and Runx3 play critical roles in iNKT cells as well (44, 45). It remains to be tested whether high P-gp expression is part of the transcriptional programming imparted by these master regulators to establish iNKT cell fate and function, potentially through modulating mitochondrial metabolism and oxidative stress as shown in CD4 and CD8 T cells (12, 32).

In summary, the present study uncovered P-glycoprotein expression/activity as an important confounding factor for using mitochondria specific tracers to characterize T cells. Memory T cells and iNKT cells that have low MTG staining signals among T cell subsets turn out to have high mitochondrial content and activity as revealed by dye-independent measurements (16). It argues that complementary methods in addition to fluorescent dyes are necessary to study mitochondria in T cells. Interestingly, other innate-like T cells, such as NK1.1^+^ γδT cells (12) and MAIT cells (46) also have high P-gp expression. While this suggests that results based on mitochondrial dye staining in these cells needs to be interpreted with caution, it also may render these cells advantageous in the context of tumors where high P-gp activity shields innate-like T cells from chemotoxicity and immune suppression (47).

## Data Availability

The datasets presented in this study can be found in online repositories. The names of the repository/repositories and accession number(s) can be found in the article/**Supplementary Material**.

## Supplementary material

Supplementary Figure 1 (Related to **Figures 1** and **3**) Higher mitochondrial content and activity in iNKT cells despite lower MTG signals. **(a, b)** MTG signals from splenic naïve (CD44^-^CD62L^+^) and memory (CD44^+^CD62L^-^) CD4 T cells stained with or without PSC833 (1μM). **(c)** Quantification of mitochondrial genome encoded genes relative to nuclear DNA/nDNA in freshly isolated splenic T cells. **(d, e)** Confocal imaging of freshly isolated splenic T cells from Dendra2 transgenic mice. **(f)** Quantification of mitochondrial volume based on Dendra2 signals as in **(d, e)**. **(g)** Average oxygen consumption rate (OCR) in sorted splenic T cells measured in a Seahorse Mitostress test. **(h)** Basal OCR, **(i)** proton leak, **(j)** maximal OCR, and **(k)** spare respiratory capacity were calculated based on **(g)**. The data shown in **(a-d, g)** are one representative experiment out of three independent experiments. The data shown in f and h-k are pooled from three independent experiments. ns: non-significant, *p < 0.05, **p < 0.01, ***p < 0.001, ****p < 0.0001, paired Student’s t test in **(b)**, unpaired Student’s t test in **(c, f, h-k)**.
