# Supplementary figures and images for: P-glycoprotein expression skews mitochondrial dye measurements in T cells
Source: Front Immunol. 2025 Jun 18;16:1560104. doi: 10.3389/fimmu.2025.1560104 (PMC12213397; doi:10.3389/fimmu.2025.1560104)

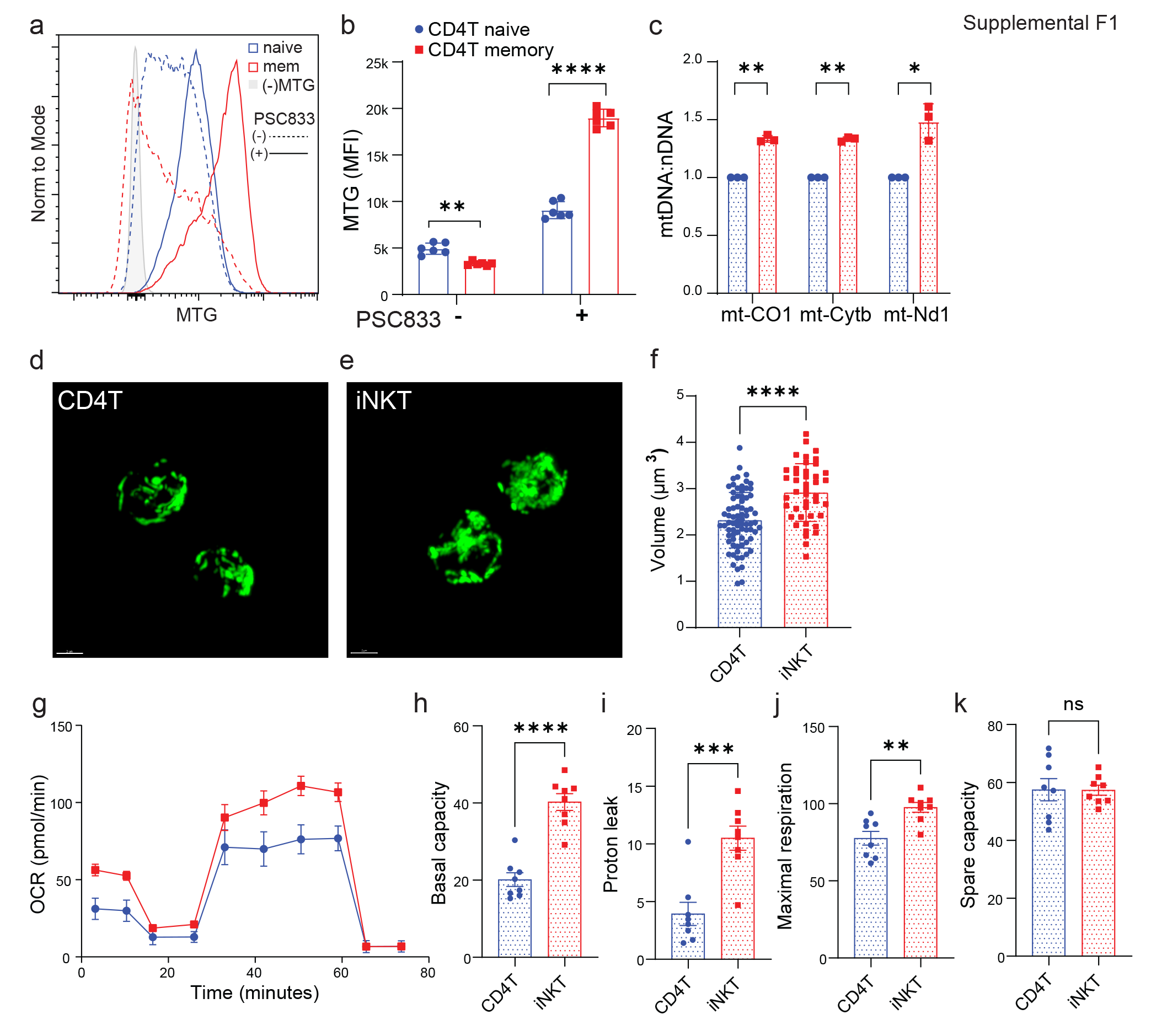

Supplement: Supplementary Figure 1 — (Related to Figures 1 and 3 ) Higher mitochondrial content and activity in iNKT cells despite lower MTG signals. (a, b) MTG signals from splenic naïve (CD44-CD62L+) and memory (CD44+CD62L-) CD4 T cells stained with or without PSC833 (1μM). (c) Quantification of mitochondrial genome encoded genes relative to nuclear DNA/nDNA in freshly isolated splenic T cells. (d, e) Confocal imaging of freshly isolated splenic T cells from Dendra2 transgenic mice. (f) Quantification of mitochondrial volume based on Dendra2 signals as in (d, e). (g) Average oxygen consumption rate (OCR) in sorted splenic T cells measured in a Seahorse Mitostress test. (h) Basal OCR, (i) proton leak, (j) maximal OCR, and (k) spare respiratory capacity were calculated based on (g). The data shown in (a-d, g) are one representative experiment out of three independent experiments. The data shown in f and h-k are pooled from three independent experiments. ns: non-significant, *p < 0.05, **p < 0.01, ***p < 0.001, ****p < 0.0001, paired Student’s t test in (b), unpaired Student’s t test in (c, f, h-k). [file Image1.tif]
